# The M6A methyltransferase METTL3 promotes the development and progression of prostate carcinoma via mediating MYC methylation

**DOI:** 10.7150/jca.42338

**Published:** 2020-03-25

**Authors:** Yan Yuan, Yang Du, Lei Wang, Xiuheng Liu

**Affiliations:** Department of Urology, Renmin Hospital of Wuhan University, Wuhan 430060, Hubei, P.R. China.

**Keywords:** N6-methyladenosine, prostate carcinoma, METTL3, MYC

## Abstract

N6-methyladenosine (m^6^A) is the richest modification in mammalian messenger RNAs (mRNAs), and exerts key roles in many biological processes, including cancer development, whereas its roles in prostate carcinoma (PCa) remain to be unclear. Here, we found that m^6^A modifications are increased in PCa and methyltransferase-like 3 (METTL3), but not other major m^6^A modification genes including METTL14, fat mass and obesity-associated protein (FTO) and AlkB homolog 5 (ALKBH5), was the major dysregulated gene associated with abnormal m^6^A modification. In addition, METTL3 up-regulation acted as a poor prognostic factor for overall survival and disease-free survival in PCa patients. Knockdown of METTL3 significantly inhibited PCa cells proliferation, migration, and invasion. In addition, over-expression of METTL3, but not its catalytic mutant form, significantly promoted PCa cells growth and progression. Mechanistically, we revealed that METTL3 enhanced MYC(c-myc) expression by increasing m^6^A levels of MYC mRNA transcript, leading to oncogenic functions in PCa. Importantly, PCa cells growth and progression inhibition by METTL3 knockdown were restored through over-expression of MYC. Our results uncovered a METTL3/m^6^A/MYC axis and provided insight into the mechanisms of PCa progression.

## Introduction

In the USA, prostate carcinoma (PCa) is the most prevalent cancer and the second leading cause of cancer deaths in the men in 2019 1. A high prevalence and mortality of PCa are also observed in European countries 2. While a large number of successful studies have been carried out on the pathogenesis of PCa, the molecular mechanisms underlying prostate carcinogenesis still not well-understood 3. It is well known that PCa is triggered by genetic and/or epigenetic disorders 4. Moreover, abnormal DNA methylation patterns are frequently reported in PCa and are known to universally affect genes involved in cancer-related processes 5,6.

Similar to DNA methylation, RNA methylation is catalyzed by methyltransferases to transfer the methyl from S-adenosylmethionine to RNA specific methylation loci 7. RNA methylation has appeared as a pervasive phenomenon and a key post-transcriptional regulator 7. "Epitranscriptomics" has been used to term this new layer of regulation 7. The most widespread RNA methylation, N6-methyladenosine (m^6^A), exists in an approximate quarter of transcripts at the genome-wide level and is enriched near stop codon, 3′ untranslated terminal regions, and within long internal exons 8. RNA m^6^A modification affects RNA splicing, transcription, stability, processing, metabolism, and translation into protein 9. RNA m^6^A is mainly imprinted by the RNA methyltransferases (METTL3, METTL14) (writers), and is erased by the demethylases (FTO, ALKBH5) (erasers) 9. Aberrant RNA methylation in different types of cancer frequently enhances oncotrascription and oncoprotein expression, which lead to cancer cell growth, development, and tumor initiation 10.

However, the role of m^6^A, “writers”, and “erasers” in PCa remains to be illuminated. Thus, our study strived to indicate the function of m^6^A modification in PCa, and ascertained the potential molecular pathways by which the m^6^A modification impacts the progression of PCa.

## Materials and methods

### Clinical specimens

Six pairs of fresh tissues, human PCa tissues and corresponding adjacent normal tissues were separately gathered from patients undergoing radical prostatectomy at the Department of Urology, Renmin Hospital of Wuhan University (Wuhan, China). A total of 84 paraffin-embedded PCa specimens and 32 paraffin-embedded corresponding adjacent normal specimens were collected from the Department of Pathology. The clinicopathological characteristics of the patients were summarized. Written informed consent was obtained from all patients before the study. The study was approved by the Institutional Research Ethics Committee of Renmin Hospital of Wuhan University.

### Immunohistochemistry (IHC)

IHC was performed as described previously 11. IHC was performed on clinical specimens to evaluate METTL3 and MYC protein expression. The specimens were disposed of the following primary antibodies: METTL3 and MYC antibody (Proteintech, China). Stained samples were scored for the percentage of positive cells (PP) and staining intensity (SI) 12. PP was scored into four categories: 0 (0% positive cells), 1 (< 33%), 2 (33% to 66%), 3 (> 66%). SI was also scored into four categories: 0 (negative), 1 (weak), 2 (moderate), 3 (strong). The IHC score was calculated by multiplying PP and SI score. Patients with different scores were divided into low- (0-4) and high-expression (6-9) groups.

### Cell culture

Cells were obtained from the American Tissue Culture Collection (ATCC). Human PCa cell lines PC3, DU145, LNCaP, C42 were cultured in RPMI-1640 medium (Gibco, USA) supplemented with 10% FBS (Gibco, USA), 100 units/ml penicillin and 100 mg/ml streptomycin. Human prostatic epithelial cell RWPE-1 were maintained in Keratinocyte Serum-Free Medium (K-SFM) supplemented with human recombinant epidermal growth factor (EGF) and bovine pituitary extract (BPE). All cells were cultured at 37 °C with 5% CO2.

### Quantitative real-time PCR (qRT-PCR)

Total RNAs were extracted from clinical tissues or cultured cells with TRIzol (Thermo Fisher Scientific, USA). RNA was subjected for the synthesis of the first-strand cDNA with PrimeScript RT Reverse transcriptase Reagent Kit (Takara, Japan). Q-PCR was performed with the SYBR Premix Ex Taq kit (Takara, Japan) detecting method on the StepOnePlus Real-Time PCR system (Applied Biosystems, USA). GAPDH was used as a housekeeping gene. The expression volume was calculated by the 2^-ΔΔCt^ method. The primers used in this study were listed below.

METTL3 forward: 5'-CATCATCCCTGCCTCTACTGG-3'

METTL3 reverse: 5'-GTGGGTGTCGCTGTTGAAGTC -3'

MYC forward: 5'-TACCCTCTCAACGACAGCAG -3'

MYC reverse: 5'-TCTTGACATTCTCCTCGGTG -3'

GAPDH forward: 5'-CGCCTTATTCGAGAGGTGTCA -3'

GAPDH reverse: 5'-TGGGCTGTCACTACGGAAGG -3'

### Western blot

Clinical tissues or cultured cells were lysed in RIPA lysis buffer (Beyotime, China). Equal amount of protein was undergone to SDS-PAGE separation and transferred to PVDF membranes (Millipore, USA). Then, the membranes were blocked, incubated with the following primary antibodies: METTL3, MYC, and GAPDH antibody (Proteintech, China). Next, the membranes were incubated with secondary antibody (Proteintech, China). Enhanced chemiluminescence (ECL) kit (Beyotime, China) was used to analyze the membranes.

### Small interfering RNA and plasmid transfections

Small interfering RNA targeting METTL3 (si-METTL3), and negative control (NC) were purchased from RiboBo (Guangzhou, China). The METTL3 interfering sequence was 5′-GCTGCACTTCAGACGAATT-3′. Human METTL3, MYC over-expression plasmids (OE-METTL3, OE-MYC), and negative control vector (Ctrl) were purchased from GenePharma (Shanghai, China). The plasmid vectors expressing shRNA that targets human METTL3 (sh-METTL3) and the scrambled shRNA (sh-Scr) were purchased from GenePharma (Shanghai, China). The shRNA sequences were as follows: sh-METTL3: 5′-GCACTTGGATCTACGGAATCC-3′, sh-Scr: 5′-GCTTCGCGCCGTAGTCTTA-3′. Plasmids for expression of wild-type METTL3 (METTL3-WT) and methyltransferase catalytic mutant METTL3 (METTL3-Mut) were kindly provided by Dr. Jun-Ju He (Central South University, China). Transfections were performed using Lipofectamine 2000 (Invitrogen, USA) for small interfering RNA and Lipofectamine 3000 (Invitrogen, USA) for plasmid according to the manufacturer's protocols.

### Analysis of m^6^A levels by Dot blot assay

The poly(A) RNAs were extracted from clinical tissues or cultured cells and were spotted onto a nylon membrane (GE Healthcare, USA). The membranes were UV cross-linked, blocked, incubated with m^6^A antibody (Abcam, USA). The membranes then were incubated with the secondary antibody. ECL kit was used to analyze the membranes. Methylene blue was performed as loading controls.

### Cell proliferation assay

Cell proliferation rates were measured using the Cell Counting Kit-8 (CCK-8) assay (Dojindo, Japan). Briefly, C42 and PC3 cells were seeded at 1×10^3^/well in 96-well plates and incubated with the desired medium. 0h and after 24h, 48h, 72h of culture, 10 μL of CCK-8 solution was added, and the cells were incubated for 3 h at 37 °C. The absorbance at 450 nm was measured.

### Wound-healing migration assay

The 5×10^5^ C42 and PC3 cells cells were seeded into 6-well plates and cultured in complete medium. A fine pipette tip was used to scratch the confluent monolayer of cells. After incubation for 24 hours, photographs were taken to estimate closure of the gap. Wound closure was evaluated and calculated as follows: wound closure = (area of gap [0 h] - area of gap [24 h])/area of gap (0 h).

### Cell invasion assay

Cell invasion assay was performed in a 24-well Transwell™ (Corning, USA). The upper chamber surface of the filter was coated with Matrigel (Corning, USA). The 2×10^4^ C42 and 3×10^4^ PC3 cells were prepared with serum-free and loaded into the upper chamber. Medium containing 20% FBS was added to the bottom chamber. After 24h incubation, the cells were stained with 0.1% crystal violet, and quantified by counting the total number of cells.

### Gene-specific m^6^A qPCR

Gene-specific m^6^A qPCR was performed as described previously 13 and to assess the relative expression level of the MYC mRNA in m^6^A antibody immunoprecipitation (IP) samples and input samples. Briefly, RNA immunoprecipitation using an m^6^A antibody was done and the m^6^A+MYC mRNA level was finally determined by qRT-PCR using the following primers.

MYC forward: 5'-TACCCTCTCAACGACAGCAG -3'

MYC reverse: 5'-TCTTGACATTCTCCTCGGTG -3'

### Statistical analysis

Each experiment was conducted in triplicate. GraphPad Prism 7.0 (San Diego, USA) and SPSS 18.0 (IBM, USA) software were used for statistical analysis. Statistical significance was accepted for P-values < 0.05, and results are represented as *P < 0.05; **P < 0.01; ***P < 0.001 and not significant (ns).

## Results

### The abnormal upregulation of m^6^A modification in prostate carcinoma

**Figure 1:**
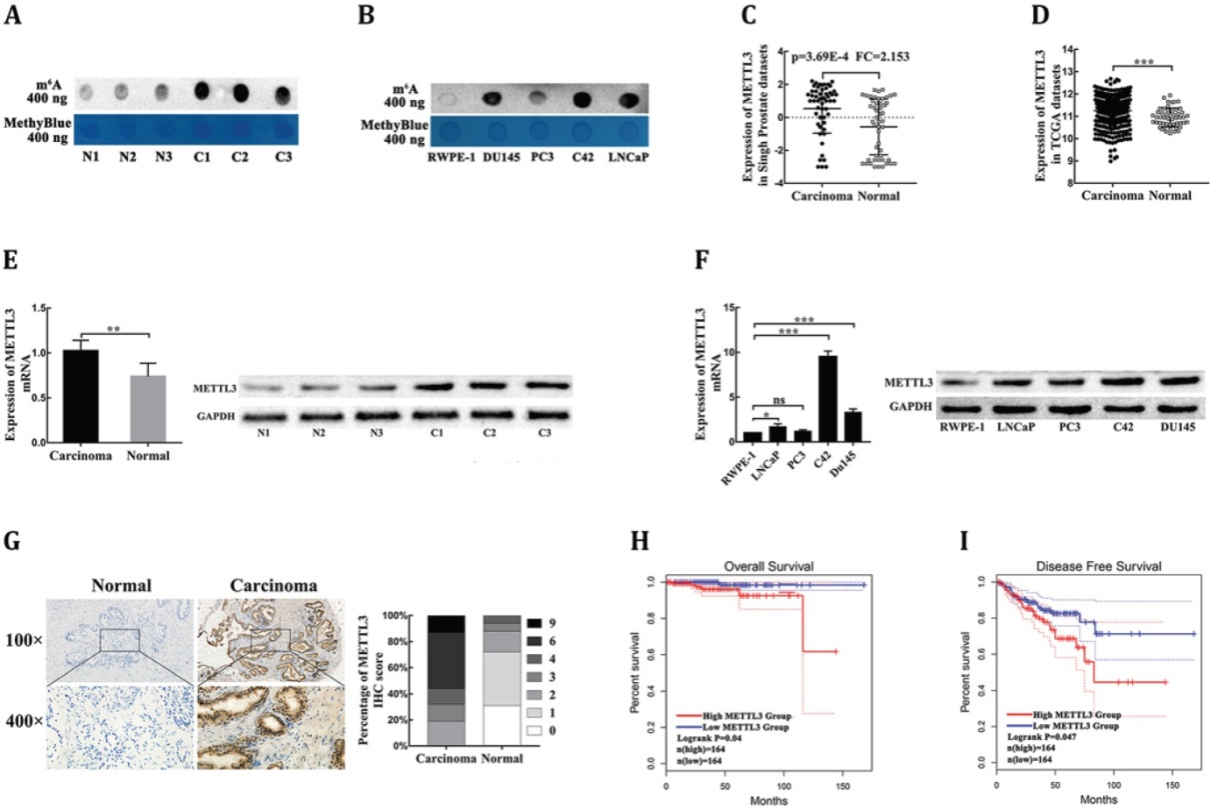
** METTL3 is responsible for the aberrant m6A modification and serves as a prognostic factor in PCa.** (**A**) The m6A contents of poly(A) RNAs in clinical PCa tissues compared with corresponding adjacent normal tissues. (**B**) The m6A contents of poly(A) RNAs in human four PCa cell lines compared with human prostatic epithelial cell (RWPE-1). (**C-D**) The mRNA levels of METTL3 in PCa tissues and normal prostate tissues were extracted from the Singh prostate dataset (C) and the TCGA database (D). (**E**) The mRNA and protein levels of METTL3 were detected in clinical PCa tissues compared with corresponding adjacent normal tissues. (**F**) The mRNA and protein levels of METTL3 in four PCa cell lines compared to human prostatic epithelial cell (RWPE-1). (**G**) The expression of METTL3 was detected by IHC assays in clinical 84 PCa specimens and 32 adjacent normal specimens. The percentage of staining gradations of METTL3 in clinical PCa specimens and adjacent normal specimens were shown. (**H-I**) The prognostic significance of METTL3 expression about overall survival (H) and disease-free survival (I) was analyzed using the GEPIA online database.

To explore the potential role of m^6^A modification in PCa, we first examined the m^6^A levels of the total mRNAs in PCa tissues, adjacent normal tissues, human PCa cell lines, and human prostatic epithelial cell. Using the dot blot assay, we found that m^6^A levels were increased in PCa tissues compared with corresponding adjacent normal tissues (Fig. 1A). In addition, m^6^A levels in the human prostatic epithelial cell were obviously reduced compared with human PCa cell lines (Fig. 1B). Together, these data indicated that m^6^A levels were increased in PCa.

### METTL3 is responsible for the aberrant m^6^A modification and serves as a prognostic factor in prostate carcinoma

To investigate the dysregulation of the key m^6^A methyltransferases and demethylase, the ONCOMINE database (https://oncomine.org/resource/main.html) was used to analyze the mRNA levels of four major m6A modification genes in PCa tissues and normal prostate tissues. Under the strict statistic criteria: P-value< 0.001 and |fod change|≥ 2, only METTL3 was found to be significantly dysregulated in sole microarray dataset: Singh prostate (Fig. 1C, Supplementary Table S1) 14. We further confirmed that METTL3 expression was significantly elevated in PCa tissues compared with normal tissues in the Cancer Genome Atlas (TCGA) datasets (https://portal.gdc.cancer.gov/) (Fig. 1D). To further determine the expression levels of METTL3 in PCa, we performed RT-qPCR and Western blot analysis of METTL3 expression in the PCa tissues and the corresponding adjacent normal tissues. The results showed that METTL3 was upregulated in the PCa tissues compared with normal tissues (Fig. 1E). Higher expression of METTL3 was also observed in the PCa cell lines compared with that in the human prostatic epithelial cell (Fig. 1F). To validate the up-regulated protein level of METTL3, we performed the IHC staining assay to detect the protein expression level of METTL3 in 84 clinical human PCa specimens and 32 corresponding adjacent normal specimens. METTL3 expression was significantly higher in the PCa samples than the normal samples (Fig. 1G). The association between METTL3 expression in the PCa and clinical-pathological characters was further studied. A significant positive association between METTL3 expression was observed with tumor stage and metastasis (Table 1). No significant association was observed between METTL3 expression and other features, such as patient age and Gleason score (Table 1). Next, the GEPIA database (https://gepia.cancer-pku.cn/index.html) was used to explore the prognostic significance of METTL3 expression in overall survival (OS) and disease-free survival (DFS) of PCa patients. The survival curves revealed that high mRNA expression of METTL3 was associated with worse OS (Fig. 1H) and worse DFS (Fig. 1I). Collectively, these results indicated that METTL3 was the main factor involved in aberrant m^6^A modification and was identified as a prognostic factor in PCa.

### METTL3 promotes prostate carcinoma cells proliferation, migration and invasion dependent on its m^6^A methyltransferase activity

**Figure 2:**
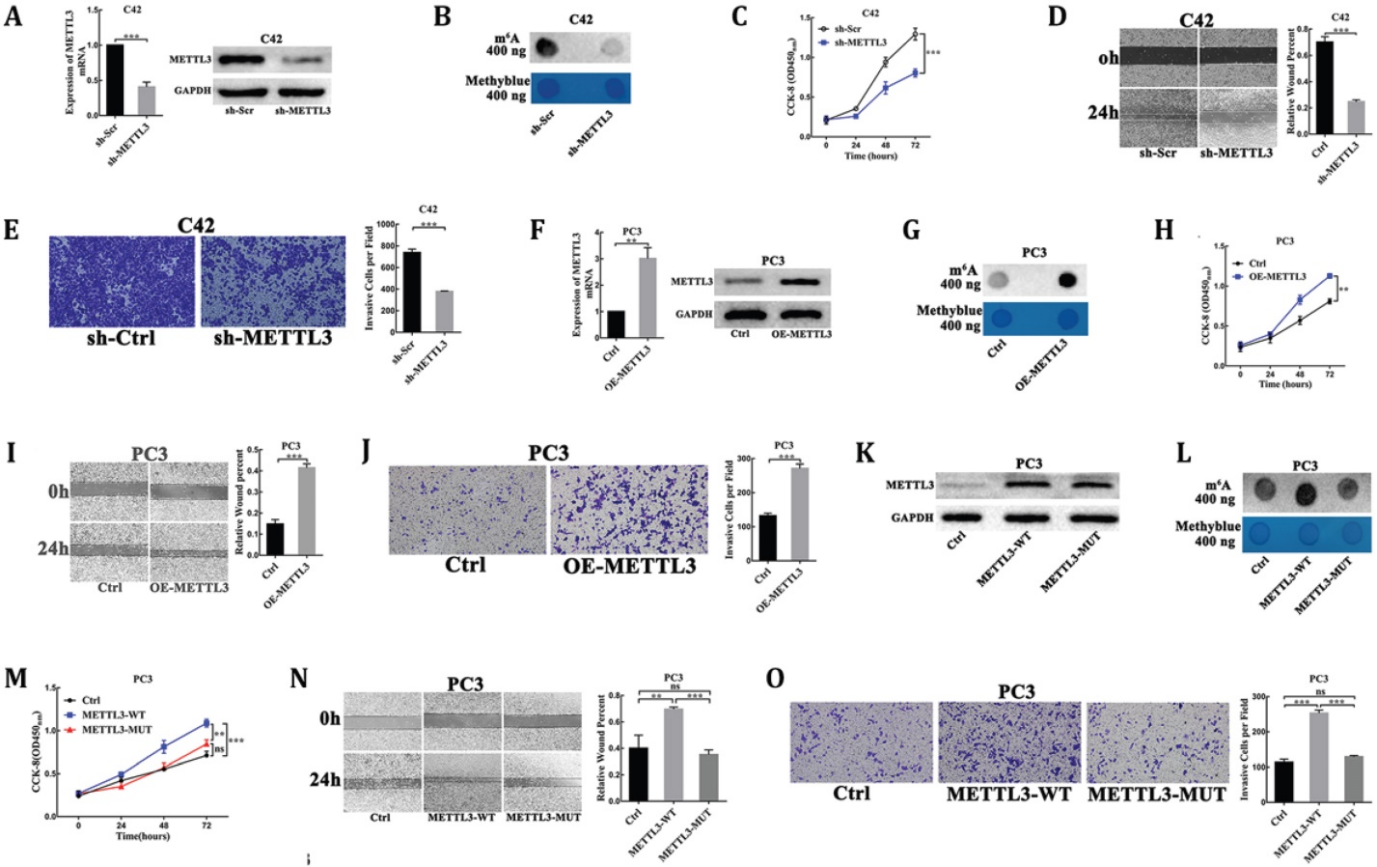
** METTL3 promoted PCa cell proliferation, migration, and invasion dependent on its m6A methylase activity.** (**A**) The interference efficiency of METTL3 in C42 cells. (**B**) The effect of METTL3 knockdown on the m^6^A contents of poly(A) RNAs in C42 cells. (**C-E**) The effect of METTL3 knockdown on C42 cells proliferation (C), migration (D), invasion (E). (**F**) The over-expression efficiency of METTL3 in PC3 cells. (**G**) The effect of METTL3 over-expression on the m^6^A contents of poly(A) RNAs in PC3 cells. (**H-J**) The effect of METTL3 over-expression on the PC3 cells proliferation (H), migration (I), invasion (J). K: Western blot confirmation of wild-type of METTL3 and catalytic mutant form in PC3 cells. L: The effect of wild-type METTL3 and catalytic mutant METTL3 on the m^6^A contents of poly(A) RNAs in PC3 cells. (M-O): The effect of wild-type METTL3 and catalytic mutant METTL3 on the PC3 cells proliferation (M), migration (N), invasion (O).

To explore the oncogenic function of METTL3 in PCa, we evaluated the cellular functions of METTL3 by loss- and gain-of-function studies. First, we used plasmid-mediated scrambled (sh-Scr) and METTL3-targeting shRNA (sh-METTL3) to deplete METTL3 in C42 cells. The interference efficiency of METTL3 was evaluated by RT-qPCR and Western blot analysis (Fig. 2A). As METTL3 is the main catalytic component in the RNA m^6^A methyltransferase complex, we examined the effect of METTL3 knockdown on the m^6^A levels of poly(A) RNAs by dot blot assay. Our results showed the reduced m^6^A level of poly(A) RNAs isolated from the METTL3 knockdown cells, as compared with the corresponding control cells (Fig. 2B). The results of the CCK8 assay showed that METTL3 depletion substantially decreased the rate of cell proliferation (Fig. 2C). We sought to uncover whether METTL3 could affect PCa cells migration and invasion. We used Wound-healing assay and Matrigel-coated transwell assay to detect the migratory or invasive potential of METTL3 knockdown or control cell. METTL3 depletion markedly reduced the migration and invasion of PCa cells (Fig. 2D, E).

To further confirm the oncogenic function of METTL3, over-expression of METTL3 plasmids (OE-METTL3) was transduced into PC3 cells. The over-expression efficiency of METTL3 was evaluated by RT-qPCR and Western blot analysis (Fig. 2F). The results showed the increased m^6^A levels of the total mRNAs isolated from the over-expression METTL3 cells compared with the control cells (Fig. 2G). The CCK-8 assay also showed that the rate of cell proliferation was significantly higher in the over-expression METTL3-expressing cells than in the control cells (Fig. 2H). The migratory and invasive abilities were significantly increased in the over-expression METTL3 cells compared with corresponding control cells (Fig. 2I, J).

Moreover, plasmids for expression of wild-type METTL3 (METTL3-WT) and catalytic mutant METTL3 (METTL3-MUT) were transduced into PC3 cells. As a result, both transfection of wild-type METTL3 and catalytic mutant METTL3 led to a significant increase at METTL3 protein levels (Fig. 2K). However, the expression of wild-type METTL3, but not the catalytic mutant METTL3, increased m^6^A levels of the total mRNAs isolated from the transfected cells and promoted cells proliferation, migration, and invasion (Fig. 2L-O).

These experiments suggested that METTL3 played an oncogenic role dependent on its m^6^A methylase activity in PCa.

### MYC is identified as a target of METTL3-mediated m^6^A modification in prostate carcinoma

**Figure 3:**
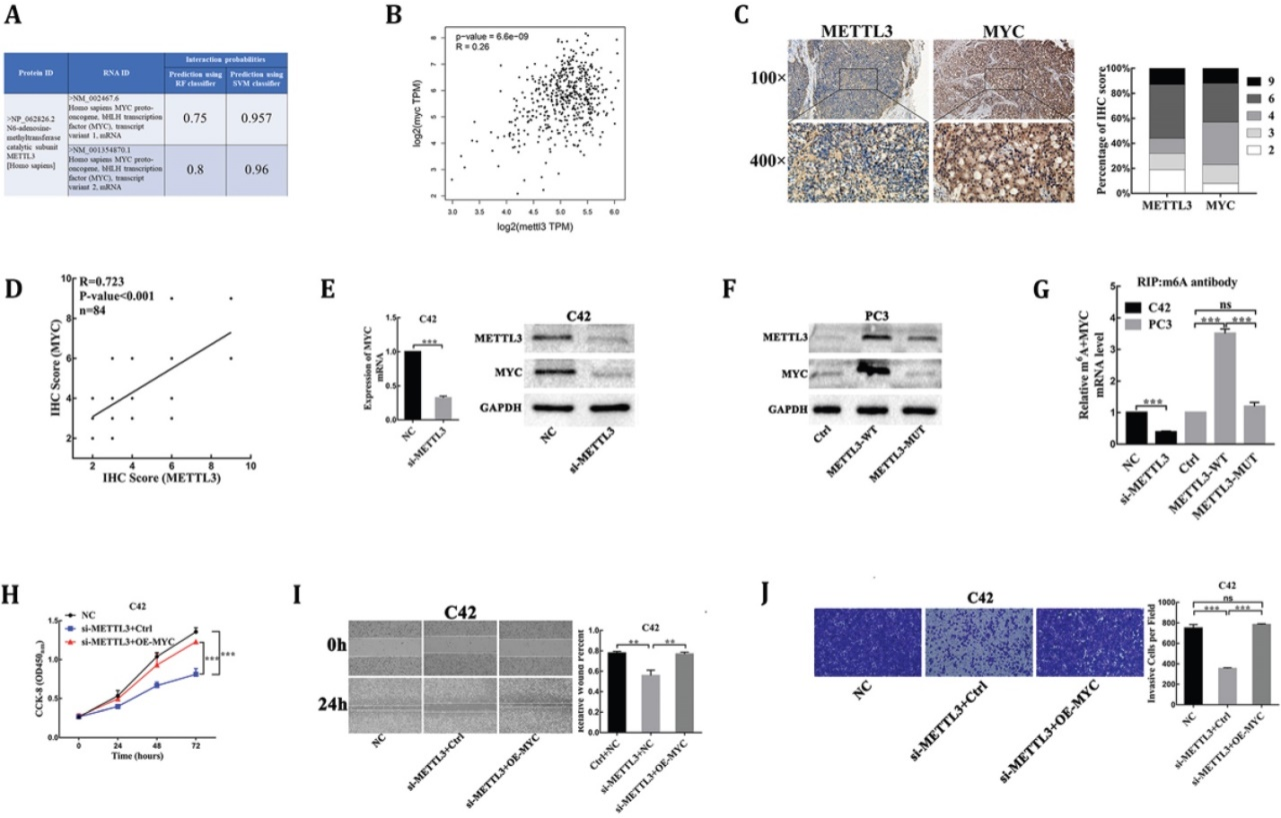
** MYC is identified as a target of METTL3-mediated m6A modification in PCa.** (**A**) Bioinformatics predicted the interaction between METTL3 protein and MYC RNA in homo sapiens (http://pridb.gdcb.iastate.edu/RPISeq/index.html). (**B**) The correlation between METTL3 and MYC using TCGA database (http://gepia.cancer-pku.cn/). (**C**) The expression of METTL3 and MYC was detected by IHC assays in clinical 84 PCa specimens. The percentage of staining gradations of METTL3 and MYC in clinical 84 PCa specimens was shown. (**D**) The correlation between METTL3 and MYC using IHC assays in clinical 84 PCa specimens. (**E**) The effect of METTL3 knockdown on the expression of MYC in C42 cells. (**F**) The effect of wild-type METTL3 and catalytic mutant METTL3 on the protein level of MYC in PC3 cells. (**G**) Gene-specific m6A qPCR validation of mRNA transcript level changes of MYC in C42 cells infected with NC or si-METTL3 and PC3 cells infected with Ctrl, METTL3-WT, or METTL3-MUT. (**H-J**) MYC over-expression rescued METTL3 knockdown-mediated inhibition of C42 cells proliferation (H), migration (I), invasion (J).

MYC(c-myc) had been discovered as an mRNA target of METTL3-mediated m6A modification by high-throughput RNA-Seq and m6A-Seq in different types of cancer 15,16. The RNA-Protein Interaction Prediction (RPISeq) database (http://pridb.gdcb.iastate.edu/RPISeq/index.html) was developed to address the need for reliable computational methods for predicting RNA-protein interactions. RPISeq predictions are based on Random Forest (RF) or Support Vector Machine (SVM) classifiers trained and tested on 2 non-redundant benchmark datasets of RNA-protein interactions, RPI2241 and RPI369, extracted from PRIDB, a comprehensive database of RNA-protein complexes extracted from the PDB. Interaction probabilities generated by RPISeq range from 0 to 1. In performance evaluation experiments, predictions with probabilities > 0.5 were considered “positive,” i.e., indicating that the corresponding RNA and protein are likely to interact. The results showed that METTL3 protein interacted with MYC mRNA in homo sapiens (Fig. 3A). Moreover, the deregulated expression of MYC proto-oncogene played a key role in PCa 17, so we analyzed whether the mRNA methylase activity of METTL3 was critical for its regulation of MYC expression in PCa. First, we analyzed and established a positive correlation between METTL3 and MYC mRNA expression in PCa tissues through the TCGA database (https://gepia.cancer-pku.cn/index.html) (Fig. 3B). Next, to validate their correlation, we performed the IHC assay to detect the protein levels of MYC and METTL3 in our 84 clinical human PCa specimens. Our results showed that samples with higher METTL3 expression were frequently associated with higher MYC level and vice versa (Table 1, Fig. 3C). Consistently, a significant positive correlation between MYC and METTL3 protein levels was observed in our clinical human PCa specimens (R=0.723, P<0.001) (Fig. 3D). Further, small interfering RNA targeting METTL3 (si-METTL3) and a negative control (NC) were transduced into C42 cells. RT-qPCR and Western blot analysis showed that the mRNA and protein expression of MYC were significantly lower in METTL3-silenced cells than negative control cells (Fig. 3E). By contrast, we showed that over-expression of wild-type METTL3 but not the catalytic mutant METTL3 significantly increased MYC protein expression (Fig. 3F). By gene-specific m^6^A qPCR validation, we showed that METTL3 silencing and forced expression of wild-type METTL3 reduced and increased the methylated MYC mRNA levels, respectively (Fig. 3G). However, when the METTL3 with methyltransferase catalytic mutant was overexpressed, the methylated MYC mRNA levels could not be affected (Fig. 3G). Meanwhile, over-expression of MYC was sufficient to rescue the inhibitory effect of METTL3 knockdown on C42 cells proliferation, migration and invasion (Fig. 3H-J), suggesting that MYC is the functional targets of METTL3 in PCa. Collectively, these findings demonstrated that MYC was identified as a functional target of METTL3-mediated m^6^A modification in PCa.

## Discussion

RNA epigenetics has become an interesting field of cancer research recently. M^6^A is the most widespread and critical biochemical modification for human RNA, and has been confirmed as a key regulator in human malignant diseases 18. More and more evidence suggests that the “writers,” “erasers,” and “readers” of m^6^A exert an important role in the cancer initiation and development 10. Due to the differences of target RNA of m^6^A modification, loci that m^6^A imprinted on RNA, “readers” identifying these m^6^A modifications, and types of cancer, roles of m^6^A in cancer growth and progression generally may be disputed. For example, either the pivotal m6A writers or erasers were found to exert oncogenic functions in certain types of cancer, including Glioblastoma (GBM) and acute myeloid leukemia 10. However, the critical m^6^A writers (METTL3, METTL14) were also reported to serve as tumor suppressor in GBM and liver cancer 19,20. Therefore, investigating the precise mechanisms underlying m^6^A modification is crucial to elucidate its function in different types of cancer. Meanwhile, currently limited research directly focuses on the role of m6A in prostate cancer, and the specific function of m6A key molecules in carcinogenesis and their underlying mechanisms remain elusive.

First, to investigate quantification level of total RNA m6A methylation in PCa, we measured m6A levels in four different PCa cell lines, prostatic epithelial cell, and six pairs of PCa tissues by Dot blot. Compared with the normal control, we observed a significant increase in total RNA m6A level in PCa cells and tissues. Next, to explore the main factors of abnormal m6A methylation level in PCa, we explored four individual m6A‐related genes expression profile in the Oncomine database. In result, we first discovered the abnormal m^6^A modification in PCa and unveiled the pivotal m^6^A writer METTL3 rather than METTL14, FTO, or ALKBH5, shown abnormal expression in human PCa. Furthermore, to further validate aforementioned findings, we confirmed the expression profile of METTL3 in PCa cell lines, prostatic epithelial cell, and PCa tissues and their matched adjacent nontumors. The results exhibited that METTL3 was significantly upregulated in PCa cells and clinical specimen. Meanwhile, The TCGA data analysis of PCa patients showed that METTL3 was not only significantly upregulated in the PCa group, but also indicated that high-expression METTL3 predicted an adverse prognosis. Collectively, our findings suggested that METTL3 may play as an oncogene in the onset and progression of PCa.

METTL3, the pivotal methyltransferase of RNA m^6^A modification, was indicated involved in the initiation and development of different types of cancer, including lung cancer 21, colon cancer 21, breast cancer 22, liver cancer 23, myeloid leukemia 15 and bladder cancer 16. Then, we functionally indicated the critical role of METTL3 in enhancing PCa cells proliferation and progression via loss- and gain-of-function studies. These findings demonstrated that METTL3 was a tumor supporter in PCa.

Importantly, RNA m^6^A modification mediated by METTL3 was currently detected to regulate various mRNA in many cancers 15,16,21-23, but its target genes in PCa still to be elucidated. Therefore, through a combination of Bioinformatics and trials, we identified the critical regulator MYC oncogene as a direct target of m^6^A modification mediated by METTL3 in PCa. First, we explored the co‐expression pattern of METTL3 and MYC expression in PCa tissues, and a significant positive correlation between METTL3 and MYC expression was noted. Next, through expression profiling and Gene-specific m6A qPCR, we validated that METTL3 increased the m^6^A levels of MYC mRNA transcript, which in turn led to the upregulation of this gene expression at both mRNA and protein level. In additon, we found that the effect of METTL3 knockdown on proliferation and progression of human PCa cells was rescued through upregulation of MYC. Indeed, the strong oncogenic roles of MYC depend on causing the aberrant expression of target genes to enhance cancer cell growth and cell progression 17,24. Mechanisms of MYC dysregulation in PCa include DNA mutation 25, noncoding RNA 26, transcriptional regulation 27, post-transcriptional regulation 28, DNA methylation 6. Corresponded to the detected abnormal m^6^A modification of MYC mRNA in many cancers, including myeloid leukemia 15, bladder cancer 16, and GBM 29, aberrant MYC expression was also caused by METTL3-mediated m^6^A modification in PCa. Finally, over-expression of the catalytic mutant METTL3 failed to enhance cell proliferation and progression of PCa, suggesting that the oncogenic function of METTL3 rely on its methyltransferase catalytic activity. Moreover, we unveiled that the catalytically inactive METTL3 failed to methylate MYC mRNA transcript and enhance MYC expression. Collectively, we found that the proto-oncogene MYC is critical downstream molecule regulated by METTL3 through m6A modification, implying that the m6A modification pathway contributes to PCa occurrence and progression.

In summary, our study uncovered a critical role of RNA m^6^A modification in regulating the progression of PCa. Meanwhile, our study demonstrated for the first time that the over-expression METTL3 predicted an adverse prognosis and exerted oncogenic roles in PCa. In addition, METTL3 contributed to the proliferation and progression of PCa through upregulation-MYC, which were dependent on METTL3 catalytic activity. Our findings demonstrated that the METTL3-m^6^A-MYC axis was crucially implicated in human PCa, demonstrated that this axis may be a potential prognostic biomarker and an effective therapeutic strategy in human PCa.

## Supplementary Material

Supplementary table S1.

## Figures and Tables

**Table 1:** The association between METTL3 expression and clinical pathological characters in PCa

Clinical pathological characters	Number of cases	METTL3		P-value
		Low-expression	High-expression	
**Age (years)**				
≤70	49	22	27	0.853
>70	35	15	20	
**Gleason score**				
≤7	54	28	26	0.053
≥8	30	9	21	
**Tumor stage**				
≤ T2	32	19	13	0.026
≥T3	52	18	34	
**Lymph node or/and distant metastasis**				
Negative	74	36	38	0.049
Positive	10	1	9	
**MYC expression**				
Low	48	32	16	<0.001
High	36	5	31	
