# Supplementary material for: The M6A methyltransferase METTL3 promotes the development and progression of prostate carcinoma via mediating MYC methylation
Source: J Cancer. 2020 Mar 25;11(12):3588–95. doi: 10.7150/jca.42338 (PMC7150444; doi:10.7150/jca.42338)
Supplement: Supplementary file 1 — Supplementary table S1. [file jcav11p3588s1.pdf]

Supplementary Table S1. The ONCOMINE database was used to analyze the mRNA levels of the four major m[6]A modification genes in PCa tissues and normal prostate tissues.

Gene	Dataset	Fold change	P-value
METTL3	Arredouani Prostate	1.308	1.01E-04
	Luo Prostate 2	1.49	9.00E-03
	Wallace Prostate	1.677	5.79E-04
	Grasso Prostate	1.343	1.66E-06
	Singh Prostate	***2.153***	***3.69E-04***
	Welsh Prostae	5.89	1.10E-02
	Yu Prostate	1.1	1.90E-02
	LaTulippe Prosate	1.097	1.53E-01
	Vanaja Prostate	1.509	5.00E-03
	Liu Prostate	1.091	1.73E-01
	Varambally Prostate	1.248	1.24E-01
	Taylor Prostate 3	1.089	4.00E-02
METTL14	Vanaja Prostate	1.336	4.00E-03
	Varambally Prostate	1.053	3.29E-01
	Taylor Prostate 3	-1.049	7.91E-01
	Grasso Prostate	-1.124	9.98E-01
	Arredouani Prostate	-1.415	9.89E-01
ALKBH5	Varambally Prostate	-1.456	5.00E-04
	Tomlins Prostate	-1.275	8.90E-02
	Lapointe Prostate	-1.067	8.49E-01
	Vanaja Prostate	1.706	2.23E-01
	Arredouani Prostate	-1.396	4.70E-04
	Luo Prostate 2	-1.148	9.20E-02
	Grasso Prostate	-1.357	4.60E-04
	Taylor Prostate 3	-1.137	1.06E-04
FTO	Holzbeierlein Prostate	-1.415	9.50E-02
	Magee Prostate	-1.182	3.00E-02
	Wallace Prostate	-1.031	3.99E-01
	Singh Prostate	-1.578	5.20E-02

Tomlins Prostate	-47.591	2.63E-01
LaTulippe Prostate	-1.58	3.60E-02
Yu Prostate	-1.195	5.00E-03
Welsh Prostae	-1.659	1.13E-04
Liu Prostate	-1.077	1.80E-02
Luo Prostate 2	-2	1.57E-01
Taylor Prostate 3	1.004	5.82E-01
Vanaja Prostate	-1.099	8.40E-02
Arredouani Prostate	-1.161	3.50E-02
Grasso Prostate	-1.476	1.73E-10
Varambally Prostate	-1.267	2.00E-03
